# A Novel Anti-Cancer Therapy: CRISPR/Cas9 Gene Editing

**DOI:** 10.3389/fphar.2022.939090

**Published:** 2022-07-22

**Authors:** Xin-Zhu Chen, Rong Guo, Cong Zhao, Jing Xu, Hang Song, Hua Yu, Christian Pilarsky, Firzan Nainu, Jing-Quan Li, Xin-Ke Zhou, Jian-Ye Zhang

**Affiliations:** ^1^ Guangzhou Municipal and Guangdong Provincial Key Laboratory of Molecular Target & Clinical Pharmacology, The NMPA and State Key Laboratory of Respiratory Disease, School of Pharmaceutical Sciences and the Fifth Affiliated Hospital, Guangzhou Medical University, Guangzhou, China; ^2^ The First Affiliated Hospital, Hainan Medical University, Haikou, China; ^3^ State Key Laboratory of Medical Molecular Biology, Department of Molecular Biology and Biochemistry, Institute of Basic Medical Sciences, Peking Union Medical College, Chinese Academy of Medical Sciences, Beijing, China; ^4^ Department of Cellular and Molecular Biology, Tuberculosis and Thoracic Tumor Research Institute, Beijing Chest Hospital, Beijing, China; ^5^ Department of Biochemistry and Molecular Biology, School of Integrated Chinese and Western Medicine, Anhui University of Chinese Medicine, Hefei, China; ^6^ State Key Laboratory of Quality Research in Chinese Medicine, Institute of Chinese Medical Sciences, University of Macau, Macao, China; ^7^ Department of Surgery, University Hospital of Erlangen, Friedrich-Alexander University of Erlangen-Nuremberg (FAU), Erlangen, Germany; ^8^ Faculty of Pharmacy, Hasanuddin University, Makassar, Indonesia

**Keywords:** CRISPR/Cas9, gene editing technology, anti-cancer therapy, off-target effect, ethics

## Abstract

Cancer becomes one of the main causes of human deaths in the world due to the high incidence and mortality rate and produces serious economic burdens. With more and more attention is paid on cancer, its therapies are getting more of a concern. Previous research has shown that the occurrence, progression, and treatment prognosis of malignant tumors are closely related to genetic and gene mutation. CRISPR/Cas9 has emerged as a powerful method for making changes to the genome, which has extensively been applied in various cell lines. Establishing the cell and animal models by CRISPR/Cas9 laid the foundation for the clinical trials which possibly treated the tumor. CRISPR-Cas9-mediated genome editing technology brings a great promise for inhibiting migration, invasion, and even treatment of tumor. However, the potential off-target effect limits its clinical application, and the effective ethical review is necessary. The article reviews the molecular mechanisms of CRISPR/Cas9 and discusses the research and the limitation related to cancer clinical trials.

## Introduction

The clustered regularly interspaced short palindromic repeats (CRISPR)/-CRISPR-associated nuclease (*Cas*) system is an acquired defense system to protect organisms from invading viruses and plasmids and is widespread in various bacteria and archaea (Jinek et al., 2012; Wiedenheft et al., 2012; Hsu et al., 2014). CRISPR loci are separated by a CRISPR array, comprising unique spacers consisted of short variable DNA sequences, which are flanked by diverse *cas* (CRISPR-associated) gene (Makarova et al., 2015; Koonin and Makarova, 2019). Among them, CRISPR is a series of short direct repeats interspaced with short sequences in *E. coli* that was discovered by Japanese researchers in 1987 (Ishino et al., 1987). The *Cas* gene encodes the *Cas* protein components with putative nuclease and helicase domains (Jansen et al., 2002; Haft et al., 2005). The CRISPR-Cas immune response includes three phases: adaptation; pre-CRISPR RNA (pre-crRNA) expression and processing; and interference to protect prokaryotes from succumbing to infection (Wiedenheft et al., 2012; Hille et al., 2018; and Koonin and Makarova, 2019).

**FIGURE 1:**
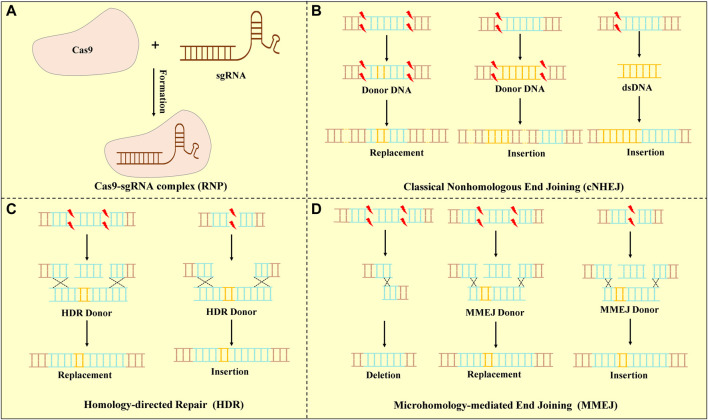
CRISPR/Cas9 system and DSB repair. **(A)** Mechanism of the CRISPR-Cas9 system. A short guide sgRNA associates with the Cas9 endonuclease to form the Cas9-sgRNA complex. Cas9 is a targeted DNA by PAM under the sgRNA. **(B)** cNHEJ : cNHEJ was the predominant pathway for repairing DSBs and was used for re-ligating broken DNA ends. Deletions or insertion mutations lead to gene frame shift mutations or premature generation of stop codes. **(C)** HDR: HDR was used a homologous DNA template to guide DSB repair. The DNA donor templates of HDR were used to insert or replace specific sequences into the genome. **(D)** MMEJ: MMEJ-mediated repair was capable of generating precise deletions between two short micro-homologous sequences (5–25 base pairs) at target loci.

The CRISPR/Cas systems are divided into two classes, which are further classified to six types and 33 subtypes that each possess signature *cas* gene (Makarova et al., 2020). The type II CRISPR/Cas9 system is mainly used in gene editing because of its simplicity, high efficacy, and ease to use (Liu et al., 2017). Cas9 is a large multifunctional protein that has two putative nuclease domains: HNH and RuvC to cleave DNA strands (Nierzwicki et al., 2021; Shams et al., 2021). The CRISPR/Cas9 system contains three core elements: the Cas9 protein, the CRISPR RNA (crRNA), and a trans-activating CRISPR RNA (tracrRNA), of which tracrRNA bears complementarity to the repeat regions of crRNA (Deltcheva et al., 2011). The Cas9 protein associates with a mature dual RNA (tracrRNA: crRNA), as a single-guide RNA (sgRNA) to target DNA cleavage. To interfere with the expression of target genes, Cas9 identifies the viral DNA sequence through a short protospacer adjacent motif (PAM) recognition that was able to select the target DNA among the genome (Sternberg et al., 2014). After the Cas9 protein binds to the target sequence, it results in double-stranded DNA breakings (DSBs) in specific regions of the genome (Frock et al., 2015). DSBs are repaired by different DNA damage repair mechanisms in cells: homology-directed repair (HDR), classical non-homologous end joining (cNHEJ), and microhomology-mediated end joining (MMEJ) (Rassool, 2003; Brinkman et al., 2018; Schep et al., 2021). Various DNA repair pathways might be used to repair each end at a DSB leading to the potential for asymmetric repair (Nambiar et al., 2022) (Figure 1). In clinical practice, these repair methods are used to achieve the purpose of relieving or even curing diseases.

DSBs are generated and repaired at specific positions in CRISPR/Cas9-mediated targeted gene segments, leading to targeted mutations because the repair process is error-prone. Though editing the targeted mutation, CRISPR/Cas9 gene editing has shown tremendous potential in oncology and has attained an encouraging achievement.

## The Research of CRISPR/CAS9 for Anti-Cancer Therapy

Cancer is the first or second leading cause of mortality and ranks as an important barrier to increasing life expectancy of the world (Bray et al., 2021; Sung et al., 2021). Tumor formation involves a variety of gene mutations and epigenetic mutations (Podlaha et al., 2012; Huang et al., 2020). Cancer genome sequencing has confirmed that there was a multitude of genes and epigenetic mutations in human tumors (Gerstung et al., 2020). At present, there are various methods to treat tumor, such as conventional cancer therapy including operation, chemotherapy and radiotherapy, molecular targeted therapy, immunotherapy, and genetic therapy. Traditional methods pose a significant challenge to patients’ tolerability and adherence due to toxicity (Mun et al., 2018). Molecular targeted drugs, one of the anti-tumor drugs, have gradually replaced traditional chemotherapeutic drugs with their advantages of high targeting and efficacy, which have made revolutionary progress in the treatment of malignant tumors. However, during the clinical application, dramatic but short-lived tumor regressions and expenses limit the benefits (Vanneman and Dranoff., 2012). Tumor immunotherapy refers to the actively or passively tumor-specific responses to suppress cancer, including immune checkpoint blocks (ICBs), adoptive cell transfer (ACT), and tumor-specific vaccines (Zhang and Zhang, 2020). Despite immunotherapy marking the beginning of a new era in cancer, it only works in a subset of cancers and a fraction of patients with cancer respond to immunotherapy (Yang, 2015). Meanwhile, the existence of immune escape makes the effect less than expected.

The gene therapy contains zinc-finger nucleases (ZFNs), transcription activator-like effector nucleases (TALENs), and the CRISPR/Cas9 system, which knockout, insert, and mutate the targeted gene to treat cancer (Knott and Doudna, 2018). ZFNs and TALENs have been applied in editing targeted gene in the body; however, they are time-consuming and complicated (Gaj et al., 2013). Compared to ZFNs and TALENs, the CRISPR/Cas9 system is the method of choice for gene editing due to its simplicity, practicability, and application diversity. Hence, the CRISPR/Cas system is used widely. For tumor and immune cells, CRISPR/Cas9 gene editing is expected to provide a new strategy for tumor therapy.

### The Application of CRISPR/Cas9 Technology in Tumor Cells

CRISPR-Cas9 technology is used in tumor cells to overcome cancer. The tumor cell editing mainly has the following methods:

(1) Gene knockout is the simplest and most common approach that has tremendous potential in clinical trials, mainly for pathogenic genes. In 2012, it was confirmed that Cas9 could cut programmatically various DNA sites *in vitro* (Jinek et al., 2012). For example, through gene editing of *CCR5* in CD4^+^ T cells from persons who have been infected with HIV helps to combat HIV infection (Hsu et al., 2014; Tebas et al., 2014; Xu et al., 2019). Similarly, disrupting the intronic erythroid-specific enhancer for the *BCL11A* gene could increase HbF protein expression to possibly cure sickle-cell anemia (SCD) and β-thalassemia (Basak and Sankaran, 2016);
(2) Targeted insertion of DNA fragments helps to correct mutation to restore the normal sequence (Mali et al., 2013). Theodore et al. applied the strategy that inserting large DNA sequences (>1 kb) helps to correct *IL2RA* mutation in cells from patients with monogenic autoimmune disease (Roth et al., 2018);
(3) Translocation of chromosomal fragment was closely associated with tumorigenesis, for instance, modeling cancer-related chromosomal translocations was able to facilitate cancer pathogenesis research (Mani and Chinnaiyan, 2010). Irina et al. have modeled the human alveolar rhabdomyosarcoma *Pax3-Foxo1* chromosome translocation in mouse myoblast by CRISPR/Cas9, which benefits researchers to explore the mechanism of the tumorigenic process (Lagutina et al., 2015);
(4) Base editing to obtain the point mutation for the purpose of various gene editing steps, such as to monitor and screen (Zhang, 2021). As we all know, the largest class of human pathogenic mutations was the point mutation (also called single nucleotide polymorphism, SNP), so that a new technology was required to change specifically an individual base pair within a vast genome (Landrum et al., 2016; Rees and Liu, 2018). Based on the CRISPR/Cas9 system, David R. Liu’s group developed a CRISPR/Cas base editor (BE) technology, also known as cytosine base editors (CBEs), to replace specific base, for instance, convert cytidine (C) to thymine (T) or guanine (G) to adenine (A) (Komor et al., 2016). Later, adenine base editors (ABEs) were developed to convert an A·T base pair to a G·C base pair (Gaudelli et al., 2017). In short, ABEs and CBEs provide four possible changes to correct point mutations (Rees and Liu, 2018).

The tumor cell models were established to test *in vitro* therapeutic effects, explore the mechanisms of drug actions, and clarify pathogenesis as well by the CRISPR-Cas9-mediated genome editing technology (Augert et al., 2020). Recently, the research about CRISPR/Cas9 gene editing technology in human cells was popularly increasing (Wang et al., 2014). The Human Genome Project and International HapMap Project showed more comprehensive data and have attracted considerable interest from scientists to explain the relation between gene and disease, especially tumor (Bayarsaikhan et al., 2021). With the involvement of the CRISPR/Cas system in drug discovery, the combination contains the CRISPR/Cas9 system, and stem cells would be applied to simulate at least 75,000 diseases associated with human genetic variants (Ledford, 2019). By revealing the gene–gene interactions to synthetic lethality of genes by the CRISPR system, we could obtain the candidate drug. The researchers exposed myeloid leukemia KBM7 cells to the CRISPR/Cas9 system that carrying a library of more than 70,000 sgRNAs; thus, drug-resistant genes would be identified (Wang et al., 2014). It was not only a complicated process in drug discovery but also time-consuming. Due to the high-screening knockout by the CRISPR/Cas9 system, it was increasing to elucidate the function of gene. Meanwhile, the induced pluripotent stem cells (iPSCs) with unlimited self-renewal capability were able to differentiate into cells of any lineage attract, which owned a value in cell disease modeling (Wang et al., 2017). An immunodeficiency, centromeric region instability, and facial anomalies syndrome (ICF) model was edited efficiently in human-induced pluripotent stem cells (hPSCs) by using the CRISPR system (Horii et al., 2013). Based on the CRISPR/Cas9 system, Paquet et al. introduced mono- and biallelic sequence changes to hPSCs to establish the model with Alzheimer’s disease-causing mutations in amyloid precursor protein and derived cortical neurons (Paquet et al., 2016). The CRISPR/Cas9 gene editing tool was combined with the *piggyBac* transposon to effectively correct the mutation of the human B hemoglobin (*HBB*) gene in the iPSC genome of patients with β-thalassemia (Xie et al., 2014). In a study, the *Lacl* gene was mutated, and several normal genes were re-expressed, which resulted in the inhibition of bladder cancer cells (Liu et al., 2014). Scientists have also cultured hPSCs *in vitro* to perfectly modify four colorectal cancer mutation genes including *APC*, *P53*, *KRAS*, and *SMAD4* and then screened the mutant cells to construct the human colorectal cancer model to explore the specific mechanism of the intestinal stem cell of colon cancer (Drost et al., 2015). In summary, the CRISPR/Cas system is typically applied for the generation of cell models.

### The Application of CRISPR/Cas9 Technology in Animals

Animal tumor models have laid a foundation for revealing the molecular mechanism of tumorigenesis and development. Animal models were ideal carriers that effectively integrate basic, clinical tumor research, which have been widely used throughout cancer research. The disease animal models played a crucial role in drug development and therapeutic approaches as an *in vivo* tool. Animal tumor models can be classified into four categories: carcinogen-induced models (CIMs), spontaneous and induced models, genetically engineered models, and transplant models, in which transplant models were divided into orthotopic models, heterotopic tumor models, and primary patient-derived xenografts (PDXs) (Burtin et al., 2020). Gene knockout animal tumor models, referring to genetically engineered models, use the gene knockout method to remove genes to induce tumorigenesis (Takeda et al., 2019). This is an ideal model for understanding the role of a single gene or several genes in tumorigenesis. Compared to other gene editing technologies such as ZFNs and TALENs, CRISPR/Cas9 technology has the advantages of cost, efficiency and timesaving. Scientists have creatively applied the CRISPR system for studying malignant tumors and constructed multiple animal tumor models. Precise gene editing has been successful in various animals, including rat (Li et al., 2013; Wang et al., 2013), goat (Ni et al., 2014), rabbit (Honda et al., 2015; Yuan et al., 2016), dog (Zou et al., 2015), monkey (Niu et al., 2014; Chen et al., 2015), pig (Zhou et al., 2015), *C. elegans* (Friedland et al., 2013), and zebrafish (Hruscha et al., 2013; Varshney et al., 2015) (Table 1).

**TABLE 1:** CRISPR/Cas9 gene editing in animals.

Animal	Targeted gene	Mutation or disruption efficiency	Other	Reference
Rat	*Tet1*	36%	*Tet1* and *Tet2* with biallelic mutations in both genes with an efficiency of 80%	Li et al. (2013); Wang et al. (2013)
	*Tet2*	48%		
	*Tet3*	36%		
Goat	*MSTN*	32%	Potential off-target but no unwanted mutation occurred	Ni et al. (2014)
	*MSTN/Prp*	20%		
	*MSTN/BLG/PrP/NUP*	4%		
Rabbit	Tyrosinase *CR2*	3%	No off-target mutation	Honda et al. (2015)
	*GJA8*	98.7%	—	Yuan et al. (2016)
Dog	*MSTN*	Monoallelic mutation: 22.7%	Biallelic mutation: 36.4%; no mutation: 40.9%	Zou et al. (2015)
Pig	*TYR*	49.4%	Double homozygous (*PAPK2 and PINK1*): 38.1%	Zhou et al. (2015)
	*PAPK2*	66.7%		
	*PINK1*	69.9%		
Monkey	*DMD* (exon 4 and exon 46)	Mosaic mutations: 87%	—	Chen et al. (2015)
	*Ppar-γ*	10%–25%	No authentic mutation was detected	Niu et al. (2014)
	*Rag1*	23.80%		
	*NrOb1*	∼20%		
Zebrafish	*bpt1*, *bplt4*, *C9t2*, and *C9t3*	86%, heritable	Mutation rates at potential off-target sites are 1.1∼2.5%	Hruscha et al. (2013)

**FIGURE 2:**
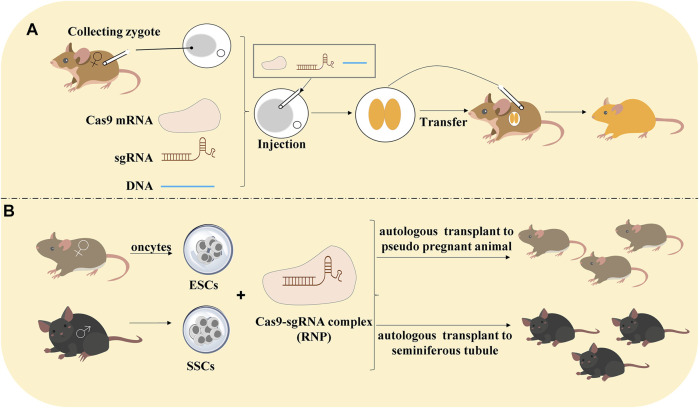
Methods of CRISPR/Cas9 technology to construct animal models. **(A)** Editing gene in embryos. Collecting the zygote that was injected with Cas9 mRNA, sgRNA, and DNA. After the zygote develops into an embryo, it would be transferred into the animals to produce generation. **(B)** Editing gene in haploid ESCs and SSCs: haploid embryonic stem cells (ESCs) are pluripotent cells generated from oocytes. CRISPR/Cas9-mediated gene editing in ESCs and autologous transplant to pseudo-pregnant animals to produce generation. Spermatogonia stem cells (SSCs) would be transfected by RNP and autologous transplant to seminiferous tubule to produce the generation.

Traditional animal models lay on the embryonic stem cells (ESCs) and HDR techniques and succeeded to establish the transgenic animal. However, it is time-consuming, and researchers spend at least 1 year to build the model. Compared with ZFNs and TALENs, CRISPR/Cas9 technology adopted three methods to build the model:

(1) Editing gene in embryos. There are 3 steps: 1) obtain zygote, 2) deliver sgRNA and Cas9 mRNA into the zygote, and 3) embryo transfer into animals to produce generation;
(2) Editing haploid embryonic stem cells (ESCs) because the haploid ESCs easily produce homozygous mutants for the generation of transgenic model;
(3) Gene editing in spermatogonia stem cells (SSCs) would be autologous transplanted to pseudo-pregnant animals (Sato et al., 2020) (Figure 2).

However, editing embryos by microinjection was too time-consuming, and the microinjection required special skill, which limited the application for high-throughput genetic analysis (Hashimoto and Takemoto, 2015). On the contrary, editing in ESCs would generate multiple knockouts and large deletions at high efficiency (Safier et al., 2020). CRISPR/Cas9-mediated gene technology in SSCs did not change the paternal imprinting pattern that displayed a great promise to treat diseases (Wu et al., 2015). From the point of view of animal studies, it is more effective for the generation of transgenic animals by CRISPR/Cas9. CRISPR/Cas9 technology is integrated into tumor cell molecular biology research, allowing for accurate and quick editing of genomes, constructing animal tumor models of gene mutation and knockout to promote comprehensive research on tumor-related genes and tumor development. The CRISPR/Cas9 system was applied not only in animals but also in insects (Shirai et al., 2022). The latest research showed that “direct parental” CRISPR (DIPA-CRISPR) was defined as a method in which the RNPs were injected into the hemocoel of females to introduce the hereditary mutation in developing oocytes and successfully applied them in cockroaches and *Tribolium castaneum*. It is an exciting breakthrough to achieve the gene editing in cockroaches due to the unique reproduction system.

### The Clinical Trial of CRISPR/Cas9 Technology

The purpose of developing new treatment methods is to achieve the purpose of preventing, alleviating, and even treating diseases. The emergence of CRISPR/Cas9 gene editing technology reflects the urgent need for treating diseases that are currently incurable, such as tumor. Applying this technology to the treatment of human diseases will bring hope to patients. In 2012, 2013, and 2015, CRISPR made the cut “breakthrough of the year,” which achieved great success such as creating a contagious gene to fight malarial infection (Hammond et al., 2016; Macias et al., 2020).

Tumor treatment is complicated and frequently accompanied by immune escape, so that it is necessary to overcome the immune escape as a crucial treatment strategy. Chimeric antigen receptor T (CAR-T) cells have been provided with tumor cell-specific antigen chimeric domains, which can activate T cells and achieve killing effect on tumor cells (Hu et al., 2021). The signaling pathway—programmed death receptor 1 (PD-1)/programmed cell death ligand 1 (PD-L1)—led to melanoma, non-small cell lung cancer (NSCLC), colorectal cancers (CRCs), bladder cancer, and renal-cell cancer if it was activated (Ott et al., 2020; O'Neil et al., 2017; Topalian et al., 2012; Stein et al., 2021; and Yi and Li, 2016). PD-1 was encoded by the *PDCD1* gene, which blocked the binding of PD-1 to its receptor PD-L1 to enhance the activation of T cells to fight cancer by improving the IFN-γ expression (Lu et al., 2020; Stadtmauer et al., 2020). Combining CRISPR/Cas9 with CAR-T cells and PD-1, the editing *PDCD1* gene in T cells was an ideal method to cure cancer (Xu et al., 2022). In 2016, the first human phase I clinical trial of CRISPR was conducted in China to therapy metastatic NSCLC patients who did not respond to chemotherapy, radiotherapy, and other therapies (Lacey and Fraietta, 2020; Lu et al., 2020). Similarly, knocking TRAC region and *CD52* gene in CAR-T cells by CRISPR/Cas9 avoided the host immune-mediated rejection for relapsed/refractory acute lymphoblastic leukemia (r/r ALL) (Hu et al., 2021). Su et al. have confirmed that targeting PD-1 in the T cells from patient with melanoma and gastric cancer resulted in the improvement of cytotoxicity of T cells, and the tumor cells were killed (Su et al., 2016). The successful practice of this gene therapy has laid the foundation for the clinical trial of CRISPR/Cas9 gene editing technology to inhibit tumor metastasis in human.

There are various genetic mutations in the process of tumor evolution such as proto-oncogene and tumor-suppressor gene. More and more mutated genes related to tumors have been identified by genome-sequencing technology. The efficient and specific gene editing function of the CRISPR/Cas9 system provides the possibility to directly target the mutated genes that cause cancer *in vivo*. As we all know, the epidermal growth factor receptor (EGFR) gene was changed in approximately 10%∼15% of NSCLC, which played an essential role in tumor progression (Koo et al., 2017). Currently, EGFR inhibitors are the first-line drugs for curing lung cancer with EGFR mutation (Janne et al., 2022). However, the development of resistance and the efficacy of drugs were limited. It was a need to develop novel tools for EGFR-mutated NSCLC, and CRISPR/Cas9 gene editing technology maybe a promising method to correct cancer-driven mutations for cancer therapy. Experiments have shown that knocking out the EGFR mutant allele (L858R) in H1975 lung cells resulted in dying of cancer cells and decreasing tumor volume . Cervical cancer was related to human papilloma virus (HPV). After targeting E6 and E7 oncogenes, tumor growth was suppressed (Khairkhah et al., 2022). However, safety and specificity of CRISPR/Cas9 need to be optimized before executing in the clinical setting. The nuclear receptor binding SET domain-containing protein 1 (NSD1) was one of the biomarkers to participate in a variety of malignancies, and human hepatocellular carcinoma (HCC) was one of them. Knocking the NSD1 gene in HCC cells led to the function of cell proliferation, migration, and invasion that were suppressed (Zhang, et al., 2019b). Targeting the reticulon 4B (Nogo-B), a negative modulator of apoptosis, was able to inhibit the ability of cell proliferation *in vitro* and tumor growth *in vivo* (Zhu et al., 2017). Based on the CRISPR/Cas9 system, it was probable to provide individualized targeted therapy, which showed potential in tumor therapy at the gene level and rises a high level. Meanwhile, the Zhang Feng’s group successfully restored vision in Leber’s congenital amaurosis type 10 (LCA10) in 2019, which showed the feasibility of CRISPR-based gene editing therapy in the treatment of genetic diseases (Maeder et al., 2019) (Table 2).

**TABLE 2:** Clinical trials of the CRISPR/Cas9 system.

Target	Therapy	Clinicaltrials.gov number	Reference
*CCR5*	HIV	NCT00842634	Tebas et al. (2014)
		NCT03164135	Xu et al. (2019)
*BCL11A*	Sickle-cell anemia (SCD) and β-thalassemia	NCT03745287	Basak and Sankaran, (2016)
		NCT03655678	Frangoul et al. (2021)
*PDCD1* (PD-1)	Non-small-cell lung cancer (NSCLC)	NCT02793856	Lu et al. (2020)
	Advanced refractory myeloma and metastatic sarcoma	NCT03399448	Stadtmauer et al. (2020)
	Relapsed/refractory acute lymphoblastic leukemia (r/r ALL)	NCT04227015	Hu et al. (2021)
	Mesothelin-positive solid tumors	NCT03545815	Wang et al. (2021)
	Metastatic colorectal cancer	NCT03174405	Stein et al. (2021)
	Prostate cancer	NCT02867345	Yi and Li, (2016)
	Bladder cancer	NCT02863913	
	Metastatic renal cell carcinoma	NCT02867332	
E6 and E7	HPV	NCT03057912	Khairkhah et al. (2022)
RPE65	Leber’s congenital amaurosis type 10 (LCA10)	NCT03872479	Maeder et al. (2019)
TTR	Transthyretin amyloidosis (ATTR amyloidosis)	NCT04601051	Gillmore et al. (2021)

## Challenges of the CRISPR/CAS9 System

As only a specific nucleic acid sequence of 20 bp was provided by using CRISPR/Cas for gene editing, its construction process was simpler and faster than ZFNs and TALENs, owing to ZFNs and TALENs must depend on the Fok I enzyme to exert. In contrast, the CRISPR technology target design was simpler and efficient, making it an ideal gene editing tool. However, it may also cause “off-target” effects, and the gene sequences should not be edited to result in unpredictable consequences. This made CRISPR more secure when used *in vivo* because once off-target occurred, it cannot be checked and corrected in a timely manner like *in vitro* experiments. The new types of Cas9, such as saCas9 and Cpf1, have been developed but cannot completely get rid of the dependence on PAM (Wang et al., 2018). This year, the newest types of Cas9-Cas9TX can inhibit the occurrence of chromosomal structural abnormalities such as chromosomal translocations and large fragment deletions in the process of gene editing and greatly improve the safety of CRISPR/Cas9 gene editing (Yin et al., 2022).

In 2018, “Gene-edited Infant” event brought a lot of controversy to the application of CRISPR at the clinical level, and direct violation of the international scientific consensus that CRISPR/Cas9 technology was not ready or appropriate for making changes to humans that could be passed on through generations until the technology matures and becomes widespread (Kofler, 2019). At this time, how to define whether accepting human beings have the right to choose their own genes in place of future generations, or whether the modified human beings can enjoy the rights of ordinary people will become a problem that needs to be pondered (Zhang et al., 2019a). The effective ethical review is the proper meaning of strengthening the ethical governance of science and technology. Therefore, it is the top priority to improve the ethical review system for human genome editing activities.

## Concluding Remarks and Future Perspectives

In the past decade, CRISPR/Cas9 gene editing technology as a strategy to therapy disease successfully entered preclinical and clinical stages. With the continuous improvement of gene editing tools and the identification of new effective targets for diseases, the clinical translation and application research of gene editing technology has been expanded. Not only in insects and plants, but also in animals and even in humans, the CRISPR/Cas9 gene editing technology proves its powerful utility.

Specific gene mutation improved tumor migration, invasion, and angiogenesis, which could be reversed by targeting editing genome. At present, the *in vivo* gene-editing based on the CRISPR/Cas system is currently being used for diseases, such as tumor and immune diseases. At present, the clinical programs are being carrying out to verify the effects of CRISPR/Cas9 and have made outstanding achievements. However, the clinical trials have been developed only involving a small number of patients and a limited follow-up, for which the further in-depth *in vivo* research studies are planned (Frangoul et al., 2021). Meanwhile, long-term safety monitoring is needed to confirm the effects and unknown adverse reactions (Frangoul et al., 2021; Gillmore et al., 2021). Developing and optimizing the Cas9-based gene editing should promote the technology forward to therapeutic applications and offer a wide variety of treating strategies for human diseases, especially tumor.

In spite of the application of CRISPR/Cas9, it brings promise for tumor therapy associated with gene mutation; problems such as off-target and ethics need to resolved. Scientists must begin to observe the international consensus and strive to advance society positively by technology (Kofler, 2019). We still have a long way to go until the CRISPR/Cas9 technology is ready to treat cancer maturely.
